# Abnormalities of intracellular organelles in metabolic dysfunction-associated steatotic disease

**DOI:** 10.1007/s00535-025-02257-5

**Published:** 2025-05-09

**Authors:** Hidenori Kido, Eishiro Mizukoshi, Masahiro Yanagi, Li Shihui, Takuya Seike, Hidetoshi Nakagawa, Tetsumori Yamashima, Yoshitake Shiraishi, Noriyuki Ozaki, Kenichi Harada, Hikari Okada, Hisanori Goto, Kumi Kimura, Yasuhiko Yamamoto, Taro Yamashita

**Affiliations:** 1 Department of Gastroenterology, Graduate School of Medical Sciences, Kanazawa University, Kanazawa, Ishikawa Japan; 2 Department of Psychiatry and Behavioral Science, Graduate School of Medical Sciences, Kanazawa University, Kanazawa, Ishikawa Japan; 3 Department of Functional Anatomy, Graduate School of Medical Science, Kanazawa University, Kanazawa, Ishikawa Japan; 4 Department of Human Pathology, Graduate School of Medical Sciences, Kanazawa University, Kanazawa, Ishikawa Japan; 5 Information-Based Medicine Development, Graduate School of Medical Sciences, Kanazawa University, Kanazawa, Ishikawa Japan; 6 Department of Biochemistry and Molecular Vascular Biology, Graduate School of Medical Sciences, Kanazawa University, Kanazawa, Ishikawa Japan

**Keywords:** MASLD, Intracellular organelle, Transmission electron microscopy

## Abstract

**Background:**

The concept of metabolic dysfunction-associated steatotic disease (MASLD) is increasingly being recognized. The mechanisms contributing to hepatocellular injury include oxidative stress owing to mitochondrial dysfunction, endoplasmic reticulum (ER) stress owing to abnormal protein accumulation in the rough ER, and disruption of cellular homeostasis and metabolic regulation to autophagic dysfunction. However, the morphological abnormalities of these intracellular organelles remain unclear.

**Methods:**

Liver tissues from model mice of MASLD, patients with MASLD, and respective controls were subjected to histopathological examination using light microscopy, and intracellular organelles were analyzed via transmission electron microscopy (TEM).

**Results:**

In model mice of MASLD, the progression of MASLD pathology was associated with abnormalities in mitochondria, glycogen granules, and rough ER. Based on these findings, the electron microscopic observations of these intracellular organelles were classified, weighted, and evaluated in liver tissues of patients with MASLD. The electron microscopic findings were significantly relatively frequent in patients with MASLD and correlated with existing histopathological scoring.

**Conclusions:**

Using TEM, we identified characteristic abnormalities in intracellular organelles specific to MASLD. These findings contribute to the understanding of the mechanisms underlying hepatocellular injury in MASLD.

**Supplementary Information:**

The online version contains supplementary material available at 10.1007/s00535-025-02257-5.

## Introduction

The worldwide prevalence of nonalcoholic fatty liver disease (NAFLD) and its advanced form, nonalcoholic steatohepatitis (NASH), is increasing; in 2019, NAFLD had an estimated global prevalence of 25% and NASH had a prevalence of 3–5% [1]. NAFLD and NASH (NAFLD/NASH) are independent risk factors for cardiovascular and liver-related diseases and all-cause mortality [2].

The term metabolic dysfunction-associated steatotic liver disease (MASLD) has been proposed in 2020 to describe fatty liver diseases associated with systemic metabolic dysfunction [3]. The concept of MASLD is superior to that of NAFLD because the diagnostic criteria are concise and do not require the exclusion of other liver disease-related complications, and histopathological examination is not mandatory. Therefore, general healthcare providers, including primary care physicians, can easily diagnose MASLD [4]. Patients at high risk for liver disease-related death or death from other causes can be easily identified, and patients with advanced liver fibrosis can be identified more efficiently than those with NAFLD [5, 6]. However, effective pharmacotherapy for MASLD pathogenesis has not been established owing to the poorly understood mechanisms and pathology of hepatocellular damage. Therefore, a detailed understanding of MASLD pathogenesis may significantly impact the quality of life of patients by uncovering molecular mechanisms directly related to prognosis. Although histopathological examination is not required for diagnosing MASLD, histopathological evaluation of liver tissue is considered important in understanding the its pathogenesis.

The mechanisms of hepatocellular damage in NAFLD/NASH include oxidative stress resulting from abnormal lipid composition and abnormal function of mitochondrial membranes, overload of free fatty acids owing to lipid abnormalities, endoplasmic reticulum (ER) stress resulting in the accumulation of abnormal proteins without folding into normal higher-order structures in the ER, disruption of intracellular homeostasis owing to abnormal autophagy/disruption of metabolic regulation, and activation of Kupffer cells by endotoxins derived from intestinal bacteria [7–10]. These mechanisms do not occur in isolation, and each pathway is thought to be intricately involved. In NAFLD/NASH, inflammatory cell infiltration in the hepatic lobule, fatty droplet deposition in liver tissue, and hepatocellular ballooning are characteristics that are useful for diagnosis, and apoptosis of hepatocytes and appearance of Mallory–Denk bodies are considered helpful pathological diagnosis [11–13]. Liver fibrosis may be observed in advanced disease states. Although these studies based on liver pathology have provided insights into the pathogenesis of NAFLD and NASH, the changes occurring within damaged hepatocytes, i.e., intracellular organelles, remain to be fully clarified. This is a common problem in MASLD.

In this study, we observed changes in intracellular organelles, such as mitochondria, intracytoplasmic glycogen granules, and rough ER (rER), with disease progression using liver tissue of model mice. Further, we analyzed the liver tissues of patients diagnosed with MASLD using transmission electron microscopy (TEM). These characteristic findings were classified and ordered with reference to electron microscopic observations in mice and their relationship to disease activity.

## Methods

### Mouse model

Male wild-type C57BL/6 mice were purchased from Jackson Laboratories (Bar Harbor, ME, USA). The mice were fed a choline-deficient methionine-reduced amino acid high-fat diet (CDAA diet) to generate CDAA-diet, high-fat diet (HF-diet), or atherogenic high-fat diet (Ath + HF-diet) mice that are widely used as a model of NAFLD and considered to meet the criteria of MASLD [14–17]. The control mice (*n* = 5) were fed a commercial standard diet for 8 weeks, CDAA-diet mice (*n* = 5) were fed a choline-deficient and L-amino acid-defined high-fat diet with 0.1% methionine (CDAHFD; A0671302; Research Diets; New Brunswick, NJ, USA) for 8 weeks [15], HF-diet mice (*n* = 3) were fed a high-fat diet (HFD; D12492; Research Diets)[16]. Ath + HF-diet mice (*n* = 5) were fed atherogenic high fat diet (Ath + HFD; D06061403; Research Diets) for 8 weeks [17]. This study was approved by the Medical Ethics Committee of Kanazawa University (study registration number: 1065), and all procedures conformed to the Declaration of Helsinki.

### Patients and laboratory testing

Forty patients who underwent liver biopsy at the Kanazawa University Hospital between 2019 and 2024 were included in this study. The electronic medical records were, retrospectively, analyzed to evaluate patient background (age and sex), physical characteristics [height, weight, and body mass index (BMI)], blood test findings (blood count, general biochemical tests, fasting blood glucose level, and coagulation capacity test), and the presence of fatty liver on abdominal ultrasound. Ultrasound-guided liver biopsy was performed using a 16G biopsy needle, and liver tissues were analyzed using light microscopy (LM) and TEM. All patients provided informed consent to participate in this study in accordance with the Helsinki declaration and the study was approved by the Medical Ethics Committee of Kanazawa University (number: 829).

### Diagnosis of MASLD

MASLD was diagnosed based on the presence of ≥ 5% fatty deposits in liver tissue through noninvasive (e.g., abdominal ultrasonography, magnetic resonance imaging, and other imaging studies) or histopathological examinations and was confirmed if the patients met one of the following conditions: (1) Type 2 diabetes, (2) multiple metabolic risks, or (3) overweight or obesity.

Metabolic risks were defined as follows:

• Waist circumference > 90/80 cm (criterion for Asian males/females).
• Blood pressure > 130/85 mmHg or specific drug treatment.
• Plasma triglycerides > 150 mg/dL (> 1.70 mmol/L) or specific drug treatment.
• Plasma high-density lipoprotein (HDL)-cholesterol < 40 mg/dL (< 1.0 mmol/L) for males and < 50 mg/dL (< 1.3 mmol/L) for females or specific drug treatment.
• Prediabetes (fasting blood sugar levels between 100 and 125 mg/dL (5.6–6.9 mmol/L), 2-h post-load glucose levels between 140 and 199 mg/dL (7.8–11.0 mmol/L) or hemoglobin A1c of 5.7–6.4% (39–47 mmol/mol).
• Homeostasis model assessment of insulin resistance score > 2.5.
• Plasma high-sensitivity C-reactive protein level > 2 mg/L.

### Observation by LM

The tissue samples were fixed with 10% neutral buffered formalin and embedded in paraffin. The tissue Sects. (4 μm-thick) were stained with hematoxylin–eosin, Masson’s trichrome, or Prussian blue. Histological findings were evaluated by a pathologist according to the Matteoni terminology, the NAFLD activity score (NAS). Blinded evaluations were conducted by two pathologists. Because of no established index for evaluating liver histopathological findings for MASLD, the Matteoni classification and NAS criteria for NAFLD/NASH were used in this study. The Matteoni classification divides NAFLD into four categories based on pathological findings: Type 1 is simple fatty liver; Type 2 is fatty liver with inflammation; Type 3 is Type 2 with hepatocellular ballooning; and Type 4 is Type 3 with Mallory–Denk body and/or liver fibrosis. The NAS [13] classifies NAFLD activity using the degree of steatosis (0–3), lobular inflammation (0–3), and hepatocellular ballooning (0–2).

### Observation by TEM

The tissue samples were cut into 1 mm^3^ blocks and immediately fixed in 2.5% glutaraldehyde for 24 h, washed with 0.1 M phosphate-buffered saline (pH 7.4), and fixed with 1% osmium tetroxide. The samples were dehydrated with graded alcohol, immersed in propylene oxide, and embedded in epoxy resin. Thin sections were stained with toluidine blue for trimming to prepare ultrathin sections. Ultrathin Sects. (70 nm) were stained with 2% uranyl acetate followed by staining with 1% lead citrate and observed using a transmission electron microscope (H-7650; Hitachi, Tokyo, Japan). Ultrastructure was blindly evaluated by an electron microscopist.

Samples, in which liver tissue covered at least five grids and 10–20 hepatocytes could be distinctly observed, were included in the analysis. Mitochondrial morphology, glycogen granules in the cytoplasm and rER were examined. With reference to the electron microscopic findings in mouse liver tissue, the findings of human samples were categorized and ordered, and those accounting for the majority of specimens are described.

### Statistical analysis

Data are expressed as the mean ± standard deviation (SD). The differences in the clinical features and histopathological findings were evaluated using Spearman’s rank correlation coefficient. A *p*-value < 0.05 was considered significant. Differences in the patient characteristics and hematological findings were evaluated using Mann–Whitney U testing. TEM findings between MASLD and non-MASLD were evaluated using Mann–Whitney U testing. Statistical analyses were performed using EZR (Saitama Medical Center, Jichi Medical University, Saitama, Japan), a graphical user interface for R (The R Foundation for Statistical Computing, Vienna, Austria).

## Results

### Light microscopic and transmission microscopic findings in MASLD model mice

**Fig. 1:**
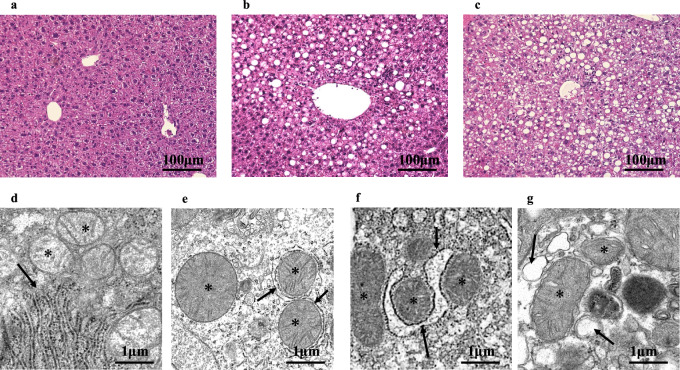
Microscopic images of liver tissue from MASLD model mice. Asterisk mitochondria; arrow, rER endoplasmic reticulum. **a**, **d** Normal-diet mice (8 weeks), **b**, **e** CDAA-diet mice (4 weeks), **c**, **f**, **g** CDAA-diet mice (8 weeks)

Liver tissue from CDAA-diet mice bred for 8 weeks showed hepatocellular ballooning, liver fibrosis, and inflammatory cell infiltration along with fatty deposits and were classified as Type 4 according to the Matteoni classification (Fig. 1a–c). In addition, this mouse model was considered to meet the diagnostic criteria for MASLD because it had fatty liver and insulin resistance, although it did not show obesity [14]. Figure 1d shows mitochondrial bilayer and cristae surrounded by rER and glycogen granules. TEM revealed circularly expanded and deformed mitochondria at 4 weeks after feeding CDAA diet (Fig. 1e). Some internal cristae were shortly truncated and formed autophagosomes. After another 8 weeks, an increase in the number of degenerated mitochondria, irregular expansion of cristae, and irregular bilayer membranes were observed (Fig. 1g). Autophagosomes were formed to envelop these structures. rER was vacuolated and exhibited an abnormal morphology (Fig. 1f). Glycogen granules in the cytosol decreased as the disease progressed. In some cases, the membrane structure was disrupted. The surrounding glycogen granules were reduced, and rER was open.

Liver tissue from HF-diet and Ath+HF-diet mice also showed hepatocellular ballooning, liver fibrosis, and inflammatory cell infiltration, along with fatty deposits, and were classified as Type 4 according to the Matteoni classification (Supplementary Fig. 1a, c). In addition, this mouse model was considered to meet the diagnostic criteria for MASLD because it had fatty liver and insulin resistance [16, 17]. LM showed that ballooning, inflammation, and fatty liver were more severe in Ath+HF-diet mice than in HF-diet mice. TEM showed that Ath + HF-diet mice exhibited severe abnormalities in mitochondria, glycogen granules, and rER, similar to those observed in CDAA-diet mice at 8 weeks (Supplementary Fig. 1d). In contrast, HF-diet mice showed only mild mitochondrial abnormalities, and their glycogen granules and rER were almost normal like those in normal diet-fed mice and CDAA-diet mice at 4 weeks (Supplementary Fig. 1b).

### Patient profiles

The patient backgrounds and hematological findings are shown in Table 1. Based on the diagnostic criteria for MASLD, 40 patients were classified into two groups: 11 in the non-MASLD group and 29 in the MASLD group. Of the non-MASLD patients, six were males and five were females. The median age of the patients was 71 years. The mean BMI was 23.5 kg/m^2^; TG was 106 mg/dL; HDL-C was 58 mg/dL; fasting blood sugar (FBS) was 115 mg/dL; alanine aminotransferase (ALT) was 36 U/L; and gamma glutamyl transpeptidase (γ-GTP) was 44 IU/L. Only one patient had CK18 > 260 U/L, which is the cut-off for diagnosis of steatohepatitis, and no patient showed liver steatosis in imaging studies. Of the patients with MASLD, 16 were males and 13 were females. The median age of the patients was 60 years. The mean BMI was 27.2 kg/m^2^; TG was 160 mg/dL; HDL-C was 45 mg/dL; FBS was 139 mg/dL; ALT was 56 U/L; and γ-GTP was 78 IU/L. Five patients had CK18 > 260 U/L and all patients showed liver steatosis in imaging studies.

**Table 1:** Patient characteristics and hematological findings

		non-MASLD, *n* = 11	MASLD, *n* = 29	*p*-value
Age, mean		68.1 (48–85)	56.8 (20–84)	0.438
Male	(*n*)	6 (55%)	16 (55%)	
BMI, mean	(kg/m^2^)	23.5	27.2	**0.023**
TG, mean	(mg/dL)	106	160	0.379
HDL-C, mean	(mg/dL)	58	45	**0.034**
FBS, mean	(mg/dL)	115	139	0.099
ALT, mean	(U/L)	36	56	0.256
γ-GTP, mean	(IU/L)	44	78	0.096
CK18 > 260 U/L	(*n*)	1	5	
Liver steatosis in imaging study	(*n*)	0	29	

P-values <0.05 were defined as statistically significant

*BMI* body mass index, *TG* triglyceride, *HDL-C* high-density lipoprotein-cholesterol, *FBS* fasting blood sugar, *ALT* alanine aminotransferase, *γ-GTP* γ-glutamyl transpeptidase, *SLD* steatotic liver disease

### Light microscopic findings in human samples

Table 2 shows the results of LM. The diagnoses in the non-MASLD group were as follows: normal in four cases, HBV (Hepatitis B virus)-related liver disease in three cases, HCV (Hepatitis C virus)-related liver disease in three cases, and other liver disease in one case. In this group, the Matteoni classification and all NAS components were normal. In contrast, all cases in the MASLD group exhibited findings consistent with SLD. In the Matteoni classification; five were diagnosed with Type 1, two with Type 2, one with Type 3, and 21 with Type 4. The breakdown of the NAS for steatosis, inflammation, and ballooning are listed in Table 2. Nine patients were classified as 0–2, 15 as 3–4, and five as ≤ 5.

**Table 2:** Light microscopic findings

		Non-MASLD, *n* = 11	MASLD, *n* = 29
Histological diagnosis in LM*, *n*	Normal	4	0
	HBV	3	2
	HCV	3	0
	SLD	0	29
	Other	1	3
Matteoni, *n*	0	11	0
	1	0	5
	2	0	2
	3	0	1
	4	0	21
NAS steatosis, *n*	0	11	0
	1	0	23
	2	0	4
	3	0	2
NAS inflammation, *n*	0	11	7
	1	0	17
	2	0	5
	3	0	0
NAS ballooning, *n*	0	11	8
	1	0	18
	2	0	3
NAS Sum, *n*	0–2	11	9
	3–4	0	15
	5-8	0	5

*LM* light microscopy, *NAS* NAFLD activity score, *HBV* hepatitis B virus, *HCV* hepatitis C virus

^*^Duplications

### TEM findings of mitochondria

**Fig. 2:**
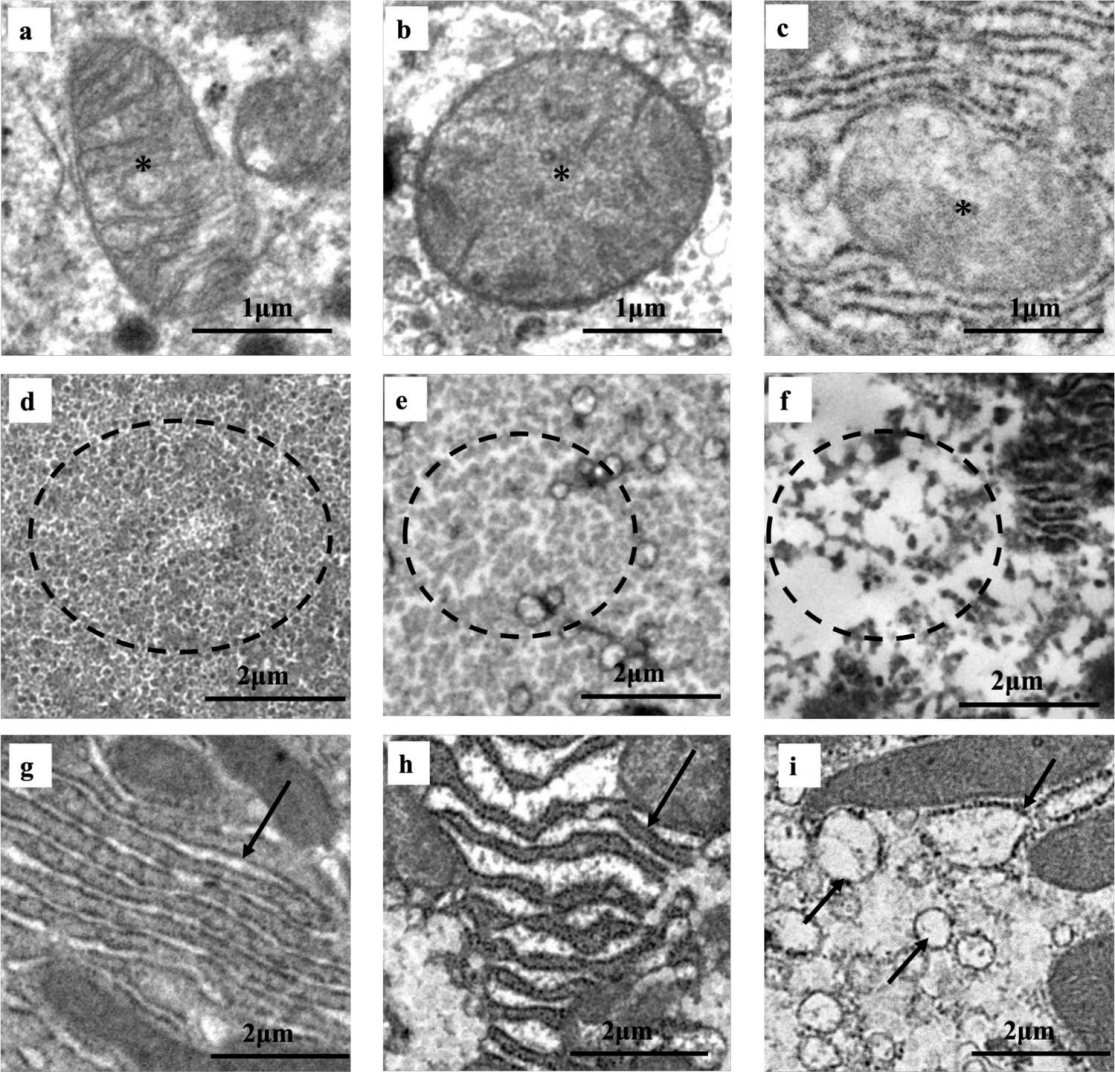
Classification of TEM findings by intracellular organelles. Asterisk mitochondria; inside dotted line glycogen granules; arrow rER. As shown below, 0 was defined as normal, 1 as mildly abnormal, and 2 as highly abnormal. TEM findings of liver tissue are summarized in Table 2. **a**–**c** TEM of mitochondria. **a** Clear membrane/normal cristae = score 0, **b** Round shape/ruptured cristae = score 1, **c** Autophagosome formation/decrease in number = score 2, **d**–**f** TEM of glycogen granules. **d** Rich = score 0, **e** Decrease in number = score 1, **f** Complete loss = score 2, **g**–**i** TEM of rER. **g** Normal = score 0, **h** Expansion/degeneration = score 1, **i** Dissolution/spherical shape (loss) = score 2

Mitochondria are intracellular organelles with a size of approximately 2 μm and exhibit a characteristic double membrane and gourd-shaped morphology. The classification of mitochondria was based on preserved morphology, characteristic double membrane structure, and distinct internal cristae. The cells with spherical morphology, ruptured internal cristae, autophagosome formation, or decreased number of mitochondria were classified as abnormal and weighted in this order (Fig. 2a–c).

### TEM findings of glycogen granules

Glycogen granules that filled the cytosol were considered normal, while those with decreased numbers and replaced by cavities were considered abnormal (Fig. 2d–f).

### TEM findings of rER

rER with a parallel-layered structure was considered normal, while that with an open lumen and wavy deformations was considered abnormal. The extremely small or laminar structures that changed to spherical shape were considered as abnormalities (Fig. 2g–i).

### TEM findings in human samples

Mitochondria were abnormal in 27 of 40 cases, with a score of 1 in 15 cases and 2 in 12 cases. The mitochondrial abnormalities were found in 4 of 11 non-MASLD individuals, with a score of 1, and most patients with MASLD (23 of 29) had abnormal mitochondria; 12 of the 23 patients had severe abnormalities with a score of 2. Glycogen granules were abnormal in 22 of 40 samples, and 7 of 22 samples had severe abnormalities with a score of 2, with all cases being MASLD. The non-MASLD patients did not have abnormal glycogen granules. Furthermore, 22 of 29 patients with MASLD had abnormal glycogen granules. Of the 40 patients, 25 had abnormal rER, with 11 having a score of 1, and 14 with severe abnormalities had a score of 2. Abnormalities of rER were identified in 4 of 11 non-MASLD patients, and 21 of 25 patients with MASLD exhibited abnormal rER morphology. The breakdown of these scores is shown in Table 3. Example of TEM findings in human samples is shown (Supplementary Fig. 2). In liver tissue from non-MASLD patients, there are numerous mitochondria with intact membrane structures, and visible cristae. Glycogen granules are abundant in the cytoplasm and the rough endoplasmic reticulum (rER) is not expanded and remains parallel. In liver tissue from MASLD patients, as seen under low magnification, the number of mitochondria is decreased, and under high magnification, the membrane structure is disrupted, and the internal cristae are no longer visible. Glycogen granules are reduced and the rER surrounding the mitochondria is expanded and degenerated. In liver tissue from MASLD patients, fat droplets are observed under low magnification.

**Table 3:** Electron microscopic findings

	Score	Non-MASLD, *n* = 11	MASLD, *n* = 29
Mitochondria score, *n*	0	7	6
	1	4	11
	2	0	12
Glycogen granule score, *n*	0	11	7
	1	0	15
	2	0	7
rER score, *n*	0	7	8
	1	2	9
	2	2	12

*rER* rough endoplasmic reticulum

### Comparison of TEM findings

**Fig. 3:**
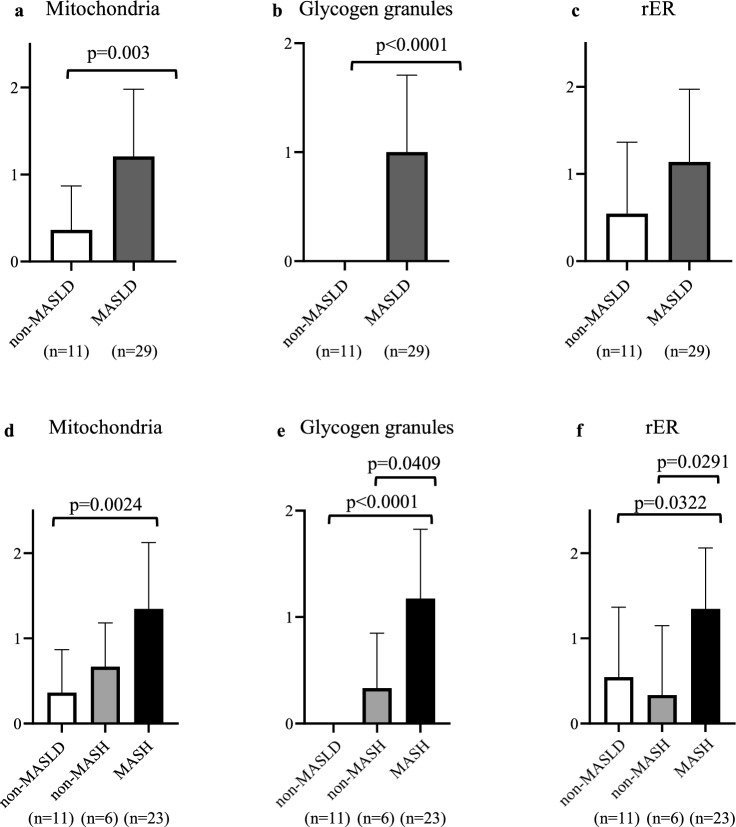
Comparison of TEM findings. **a**–**c** Comparison of TEM findings between the non-MASLD and MASLD groups. **d**–**f** Comparison of TEM findings between among the non-MASLD, non-metabolic dysfunction-associated steatohepatitis (non-MASH), and metabolic dysfunction-associated steatohepatitis (MASH) groups. The MASLD group was divided into two subgroups, non-MASH (*n* = 6) and MASH (*n* = 23) groups based on the presence or absence of hepatitis findings. The non-MASH group corresponded to simple steatosis without inflammation or fibrosis

TEM findings of liver tissues between the non-MASLD and MASLD groups were compared using the Mann–Whitney U test. Significant differences in mitochondria (*p* = 0.003), and glycogen granules (*p* < 0.0001) were observed between the two groups (Fig. 3a, b). However, no significant difference was observed in rER between the two groups (Fig. 3c). Furthermore, the MASLD group was divided into two subgroups, non-metabolic dysfunction-associated steatohepatitis (non-MASH) (*n* = 6) and metabolic dysfunction-associated steatohepatitis (MASH) (*n* = 23) groups based on the presence or absence of hepatitis findings, and an analysis was conducted. The non-MASH group corresponded to simple steatosis without inflammation or fibrosis. Comparison between the non-MASLD and non-MASH groups showed no significant differences in the scores of TEM findings (Fig. 3d–f). In contrast, comparison between the non-MASLD and MASH groups showed significant differences in the scores for mitochondria (*p* = 0.0024), glycogen granules (*p* < 0.0001), and rER (*p* = 0.0322). Additionally, comparison between the non-MASH and MASH groups showed significant differences in the scores for glycogen granules (*p* = 0.0409) and rER (*p* = 0.0291); however, no significant difference was observed in the scores for mitochondria.

### Analysis of the association between TEM and LM findings

**Fig. 4:**
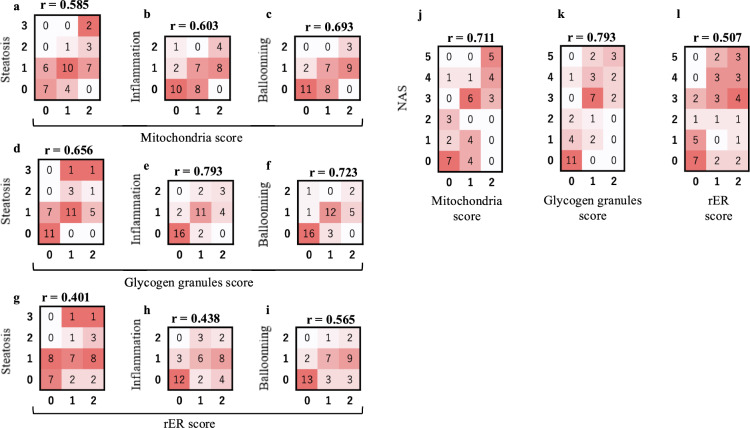
Correlation between NAS and transmission electron microscopic findings. The correlation of each index was examined using Spearman’s correlation coefficient. The correspondence among TEM findings, NAS items, and the number of patients is presented in Tables 2, 3. The number in each square indicates the number of patients who presented with the relevant findings. The intensity of the red color in each box increases with an increase in the number of individuals with each score

Next, we compared the TEM findings of human liver tissue with each item of the NAS (steatosis, inflammation, and ballooning) and the total NAS and used Spearman’s correlation coefficient to determine the correlation between these indices (Fig. 4). The mitochondrial abnormalities were strongly correlated with steatosis (*r* = 0.585, *p* = 0.0000727), inflammation (*r* = 0.603, *p* = 0.0000376), and ballooning (*r* = 0.693, *p* = 0.000000719621) (Fig. 4a–c). Abnormalities in glycogen granules were strongly correlated with steatosis (*r* = 0.656, *p* = 0.00000428), inflammation (*r* = 0.793, *p* = 0.00000000109), and ballooning (*r* = 0.723, *p* = 0.000000141) (Fig. 4d–f). Abnormalities in rER correlated with steatosis (*r* = 0.401, *p* = 0.0103), inflammation (*r* = 0.438, *p* = 0.00472), and ballooning (*r* = 0.565, *p* = 0.000146). The total NAS was highly correlated with abnormalities in mitochondria (*r* = 0.711, *p* = 0.00000027), glycogen granules (*r* = 0.793, *p* = 0.00000000106), and rER (*r* = 0.507, *p* = 0.00085) (Fig. 4j–l).

## Discussion

Liver biopsy and LM are the gold standards in diagnosing NAFLD/NASH; however, only few reports have described the characteristic findings of TEM. Moreover, reports of such findings are even rare for MASLD. Electron microscopic evaluation of the differences in endocytic organs in NAFLD and NASH has indicated that the findings in both are similar [18]. In addition to intracellular organelles, such as mitochondria, foamy cytoplasmic appearance, and lipofuscin granules, the environment outside hepatocyte, including hepatocyte-to-cell distance and bile duct diameter, has been examined, and significant difference in the diameter of mitochondria between NAFLD and NASH has been noticed; important findings, although not significant, included megamitochondria, foamy cytoplasm of hepatocytes resulting from glycogen accumulation, smooth ER dilation, and the number of lipofuscin granules [18]. In the present study, megamitochondria were observed in only one case and were not included in the analysis because of their small number and ambiguous definition. We focused on the morphology and membrane structure of mitochondria, rather than the diameter. Measurement of mitochondrial size in thin slices prepared for electron microscopy may cause discrepancies, and measuring each of the many mitochondria within a cell is clinically impractical. With reference to previous electron micrographs [18], the foamy cytoplasm of hepatocytes owing to glycogen accumulation and smooth ER dilation were considered to be the shedding of glycogen granules and lipofuscin granules were identical to the autophagosomes in this study. Glycogen is an important energy source, which maintains blood glucose levels during fasting. Under conditions of mitochondrial dysfunction, glycogenolysis may increase, thereby resulting in glycogen depletion. We noticed glycogen depletion in the TEM images. ER stress occurs in the liver of patients with NAFLD/NASH owing to factors, such as saturated fatty acids, hyperinsulinemia, and oxidative stress [19]. Furthermore, ER stress causes cellular ballooning in hepatocellular carcinoma, a characteristic finding in NAFLD/NASH [20]. In addition, an association between ER stress and NAFLD/NASH has been indicated in high-fat diet-fed MUP-uPA mice, a model that highly expresses urokinase plasminogen activator specifically in hepatocytes and is subjected to ER stress. ER stress promotes fatty acid synthesis by activating sterol regulatory element-binding protein-1c and inhibiting apolipoprotein B-100 translation and very low-density lipoprotein (VLDL) production and causes oxidative stress [21]. In this study, the opening of rER, as also noted in previous studies [19–21], indicated ER stress. Furthermore, 21 of 29 (72.4%) patients with MASLD showed morphological abnormalities, such as open lumens with wavy deformations, extremely small numbers of lumens, and laminar structures that changed to spherical structures, thereby reflecting ER stress.

The limitations of this study include a low number of observed cells and low NAS of the targeted population.

Our findings effectively illustrate the characteristics of MASLD, in which oxidative stress induces mitochondrial degeneration and dysfunction, glycogen depletion owing to disruption of the ATP synthesis pathway, and ER stress.

The TEM analyses indicated that changes in mitochondria, glycogen granules, and rER were observed significantly more frequently in liver tissue of patients with MASLD than in those of non-MASLD patients and correlated with disease activity items in the existing histopathological assessment method of NAS, suggesting that they are characteristic of MASLD. These findings may provide mechanistic insights into the abnormalities of intracellular organelles in the etiology of MASLD.

## Conclusion

Herein, we identified abnormalities in intracellular organelles characteristic of MASLD, such as abnormal mitochondrial morphology, decreased glycogen granules, and enlargement and deformation of rER. These findings contribute to our understanding of the mechanisms underlying hepatocellular injury in MASLD.

## Supplementary Information

Below is the link to the electronic supplementary material.

Supplementary file1 (DOCX 9956 KB)
