# Supplementary material for: Abnormalities of intracellular organelles in metabolic dysfunction-associated steatotic disease
Source: J Gastroenterol. 2025 May 9;60(8):990–9. doi: 10.1007/s00535-025-02257-5 (PMC12289852; doi:10.1007/s00535-025-02257-5)
Supplement: Supplementary file 1 — Supplementary file1 (DOCX 9956 KB) [file 535_2025_2257_MOESM1_ESM.docx]

**Electronic Supplementary Material**

**Supplementary Figure 1. Microscopic images of liver tissue from other MASLD model mice**

Asterisk, mitochondria; arrow, rough endoplasmic reticulum.

a and b, high fat diet-fed mice; c and d, atherogenic high fat diet-fed mice.

**Supplementary Fig2. TEM findings in human samples**

Asterisk: mitochondria, arrow: rough endoplasmic reticulum, circle: glycogen granules.

a, b TEM findings in non-MASLD patient. c,d TEM findings in MASLD patient (NAS=5).
